# A Facile Method for the Fabrication of Silver Nanoparticles Surface Decorated Polyvinyl Alcohol Electrospun Nanofibers and Controllable Antibacterial Activities

**DOI:** 10.3390/polym12112486

**Published:** 2020-10-26

**Authors:** Yan Yang, Zhijie Zhang, Menghui Wan, Zhihua Wang, Xueyan Zou, Yanbao Zhao, Lei Sun

**Affiliations:** 1 Engineering Research Center for Nanomaterials, Henan University, Kaifeng 475004, China; yy_931210@163.com (Y.Y.); wanmenghui950825@163.com (M.W.); zouxueyan@henu.edu.cn (X.Z.); zhaoyb902@henu.eud.cn (Y.Z.); 2 Ministry of Education Key Laboratory of Advanced Civil Engineering Material, School of Materials Science and Engineering, and Institute for Advanced Study, Tongji University, Shanghai 201804, China; zzj941314@163.com; 3 Henan Engineering Research Center of Industrial Circulating Water Treatment, College of Chemistry and Chemical Engineering, Henan University, Kaifeng 475004, China

**Keywords:** Ag nanoparticles, polyvinyl alcohol, electrospun nanofibers, solvothermal method, antibacterial properties

## Abstract

Polyvinyl alcohol (PVA) electrospun nanofibers (NFs) are ideal carriers for loading silver nanoparticles (Ag NPs) serving as antibacterial materials. However, it is still a challenge to adjust the particles size, distribution, and loading density via a convenient and facile method in order to obtain tunable structure and antimicrobial activities. In this study, Ag NPs surface decorated PVA composite nanofibers (Ag/PVA CNFs) were fabricated by the solvothermal method in ethylene glycol, which plays the roles of both reductant and solvent. The morphology and structure of the as-fabricated Ag/PVA CNFs were characterized by scanning electron microscopy, transmission electron microscopy, selected area electron diffraction, X-ray diffraction, UV-visible spectroscopy, and Fourier transform infrared spectroscopy. Ag NPs had an average diameter of 30 nm, the narrowest size distribution and the highest loading density were successfully decorated on the surfaces of PVA NFs, at the AgNO_3_ concentration of 0.066 mol/L. The antibacterial properties were evaluated by the methods of absorption, turbidity, and growth curves. The as-fabricated Ag/PVA hybrid CNFs exhibit excellent antimicrobial activities with antibacterial rates over 98%, especially for the sample prepared with AgNO_3_ concentration of 0.066 mol/L. Meanwhile, the antibacterial effects are more significant in the Gram-positive bacteria of *Staphylococcus aureus (S. aureus)* than the Gram-negative bacteria of *Escherichia coli (E. coli)*, since PVA is more susceptive to *S. aureus*. In summary, the most important contribution of this paper is the discovery that the particles size, distribution, and loading density of Ag NPs on PVA NFs can be easily controlled by adjusting AgNO_3_ concentrations, which has a significant impact on the antibacterial activities of Ag/PVA CNFs.

## 1. Introduction

Ag based nanomaterials are widely used in photoelectric and biological applications, due to their controllable morphology and size [1,2,3]. Especially serving as antimicrobial materials, Ag nanoparticles (NPs) exhibit broad-spectrum, superior, and long-lasting antimicrobial effects compared to synthetic and natural antibacterial materials [4,5]. Although Ag NPs have excellent antibacterial activities, self-aggregation is a major problem in their practical application [6]. Therefore, it is important to fabricate a suitable carrier that can load Ag NPs with high dispersibility. Numerous materials, such as graphene [7], hydroxyapatite [8], silica [9], and polymers [10,11,12] have been employed as the matrix material for the loading of Ag NPs, which are embedded into the interior or decorated on the surface of substrates. Among them, electrospun nanofibers (NFs) as supporting materials for Ag NPs fixation have become a hot topic in this field [13,14,15,16] due to their significant advantages, such as their free-standing ability, large specific surface area, and high porosity and permeability. There are two ways to combine Ag NPs with electrospun NFs. The first method is direct blending, in which Ag NPs are first mixed with matrix solution and then electrospun [17,18,19]. Ag NPs are implanted into NFs through this method, unfortunately, the morphology and size of NPs can hardly be controlled, since they tend to be aggregated and deformed under the influence of high voltage electric field. However, the second method of post-treatment provides a strategy to easily control the morphology, size, coverage density, and distribution of NPs on the surface of electrospun fibers, through the in-situ growth of NPs on the as-prepared electrospun NFs [20].

Most polymers are suitable for electrospinning, especially polyvinyl alcohol (PVA) [21,22,23]. PVA has been widely applied to fabricate micro/nanofibers with the advantages of biocompatibility, biodegradability, nontoxicity, and low cost. Moreover, Ag NPs can be easily loaded on the PVA surface via chemical interaction, since PVA polymer chains are abounded of hydroxyl groups. Unfortunately, PVA polymers are liable to swelling and form membranes in aqueous solution [24]. The common method to avoid swelling is chemical or physical crosslinking, such as the glutaraldehyde gas crosslinking method [25,26]. However, this post treatment method is too complicated. As such, the preparation of Ag NPs in non-aqueous solvent has become a reasonable alternative for the modification of PVA electrospun NFs and overcoming their swelling effects. In this regard, the polyol process has more advantages, including its low toxicity, being more environment friendly, and its low cost [27,28].

In this paper, we combine the electrospun and polyol solvothermal methods to fabricate Ag NPs decorated on the surface of PVA NFs with tunable size and density of Ag NPs for gaining excellent antibacterial performance. The PVA NFs were first prepared through the electrospinning process. Subsequently, Ag/PVA composite nanofibers (CNFs) were fabricated by the in-situ deposition of Ag NPs on the surface of PVA NFs via polyol solvothermal method. Herein, ethylene glycol (EG) is not only acting as a solvent, but also a reducing agent in the reaction. It is a very facile and mild method to prepare Ag NPs coated PVA NFs. Furthermore, Ag NPs are rigidly fixed on the PVA through chemical interaction, which avoid the pre-grafting modification of polymer fibers. In addition, the sizes and loading densities of Ag NPs can be conveniently tailored by regulating the concentration of AgNO_3_. The morphology and structure of the as-fabricated Ag/PVA CNFs were intensely investigated by techniques of scanning electron microscopy (SEM), X-ray powder diffraction (XRD), transmission electron microscopy (TEM), selected area electron diffraction (SAED), UV-vis spectroscopy (UV-vis), and Fourier transform infrared spectroscopy (FTIR). The antibacterial activity of Ag/PVA CNFs was evaluated by the methods of absorption, turbidity, and growth curves using the Gram-negative bacteria of *Escherichia coli (E. coli)* and the Gram-positive bacteria of *Staphylococcus aureus (S. aureus)* as target species. The significant advantage of this study is that by adjusting the concentration of AgNO_3_, the particle size and distribution density of Ag NPs on the surface of PVA NFs can be easily adjusted, so as to achieve the tunable antibacterial properties of composite nanofibers.

## 2. Materials and Methods

### 2.1. Reagents

Silver nitrate (AgNO_3_), PVA (degree of polymerization = 1750 ± 50, deacetylation degree > 98%), EG, and sodium dodecyl sulfate (SDS) were purchased from Sinopharm Chemical (Beijing, China). Nutrient agar and broth medium are both biochemical reagents (BR) and were purchased from Beijing Aoboxing Biotechnology Corporation (Beijing, China). *E. coli* (ATCC 23282) and *S. aureus* (ATCC 35696) bacterial strains were obtained from China General Microbiological Collection Center (CGMCC, Beijing, China). All the above reagents were used as received without further purification. Distilled water was used throughout the experiment.

### 2.2. Instruments and Characterization

SEM images were taken on a scanning electron microscope (FEI Nova NanoSEM 450, Thermo Fisher Scientific, Waltham, MA, USA), at the working distance of 5 mm, operation voltage of 5 kV and spot size of 3.0 nm. XRD patterns were collected on an X-ray diffractometer (BRUKER D8-ADVANCE, Bruker Co., Karlsruhe, Germany) using Cu Kα (λ = 1.5418 Å) radiation, with operation voltage 40 kV and current 40 mA, respectively. TEM images and SAED were observed on a transmission electron microscope (JEOL JEM-2100, JEOL, LTD, Akishima, Japan) at an acceleration voltage of 200 kV. UV–vis absorption spectra were obtained by using an UV-Vis spectrophotometer (PE Lambda 950, PerkinElmer Inc., Waltham, MA, USA) with a wavelength range of 300–800 nm. FTIR spectra were recorded on a Fourier transform infrared spectrometer (NICOLET AVATAR 360, Nicolet Instrument Corp., Richardson, TX, USA) with a wavenumber range of 4000–500 cm^−1^.

### 2.3. Preparation of PVA Spinning Solution

In order to obtain smooth and continuous PVA NFs, SDS was added to PVA solution to increase the surface tension of the solution to make the electrospinning process successful [29]. In detail, 3.20 g PVA and 0.008 g SDS was added into 40 mL distilled water, the solution was stirred and refluxed at 95 °C for 2 h until it became transparent. The PVA solution was stored in oven at 60 °C for the electrospinning process.

### 2.4. Electrospinning of PVA NFs

We set up the electrospinning equipment ourselves, which was described in detailed in our previous work [19]. In our previous study, we found the appropriate parameters for preparation of electrospun fibers i.e., solution concentration is 7–8%, applied electric field is 14–18 kV, feed rate is 0.010–0.015 mL/min, tip-to-collector distance is 8–12 cm, and needle type is 19–21. Herein, this optimized condition was conducted for the electrospinning of the PVA NFs. The detailed procedure is as follows: the as-fabricated 8% PVA spinning solution was filled into a 10 mL plastic syringe capped with a 20 G stainless-steel needle with a blunt end tip. The metal needle tip was performed at 17 kV high voltage, with a feed rate of 0.010 mL/min through the process. The PVA NFs were collected on the aluminum foil, which covered the collector plate and electrically grounded. The deposition time of the PVA NFs lasted for 60 min. After that, the PVA NFs were separated from the aluminum foil and maintained for further characterization.

### 2.5. Growth of Ag NPs on the Surface of PVA NFs

Following the typical procedure, 10 mg of the as-fabricated PVA NFs was cut into 1 cm × 1 cm small pieces and added into 15 mL EG with magnetic stirring for 1 h. Subsequently, 5 mL 0.066 mol/L AgNO_3_ solution dissolved in EG was poured into it and kept stirring for about 3 min. After that, the mixed solution was transferred into a 50 mL Teflon-lined autoclave to implement the hydrothermal reaction. The autoclave was kept at 160 °C for 2.5 h, and then, it was cooled down to ambient temperature naturally. The obtained Ag/PVA CNFs were washed with ethanol under ultrasonication for three times. In order to control the size and coverage density of Ag NPs on the PVA NFs surface, different concentrations (0.016, 0.033, 0.049, 0.066, 0.098, 0.132 mol/L) of AgNO_3_ solution in EG were adapted to the solvothermal precursor, while the other conditions remained the same.

### 2.6. Antimicrobial Activity Testing

The antibacterial activity of pure PVA NFs and Ag/PVA CNFs were evaluated through the methods of turbidity and absorption against *E. coli* and *S. aureus*. Glassware, suction nozzles, and culture medium were sterilized in an autoclave at a high pressure of 0.1 MPa and a temperature of 121 °C for 20 min before test. The test procedures of turbidity and absorption were conducted in accordance with our previous report [30]. Besides, the bacteria growth curves method based on the turbidity method was also employed in this study to verify the sustaining bactericidal activity. Firstly, 5 mg of Ag/PVA CNFs was immersed into a 10 mL test tube and irradiated in ultraviolet light for over 5 h. Subsequently, 40 μL bacterial suspensions (10^8^ colony forming units (CFU)/mL) were inoculated to the Ag/PVA CNFs. Then, 4 mL broth medium was injected into the tube. After that, the tube was incubated in an ATS-03M2R linear shaking bath (Shanghai Kanxin Instrument Co., Ltd.; Shanghai, China) at 37 °C, and the incubated solutions were extracted every 8 h for turbidity measurements, in which the absorbance intensities (optical density) centered at 600 nm (OD600) were recorded by using with a 725N UV–vis spectrophotometer (Aopule Instrument Co., Ltd.; Shanghai, China). As the control test, 40 μL of the bacterial suspension was injected into 4 mL of the broth medium without the addition Ag/PVA CNFs with the same operation procedure.

**Figure 1:**
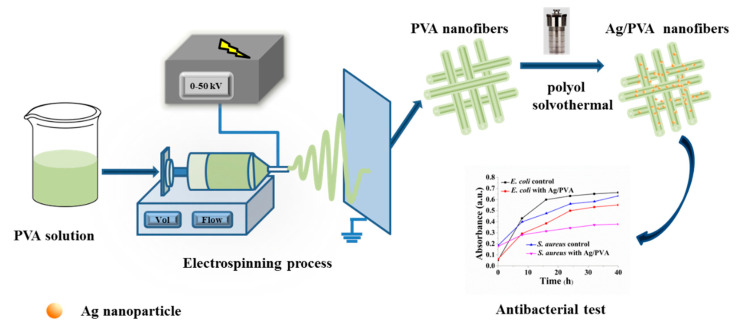
Schematic illustration of fabrication and antibacterial test for Ag/PVA composite nanofibers (CNFs) through the electrospinning and solvothermal methods.

Briefly, in this paper, we fabricated Ag/PVA CNFs through a combination of electrospinning and polyol solvothermal processing and investigated its antibacterial activates. The whole research route can be summarized by a schematic illustration, as shown in Figure 1.

## 3. Results and Discussion

### 3.1. Morphology and Structure of Ag/PVA CNFs

**Figure 2:**
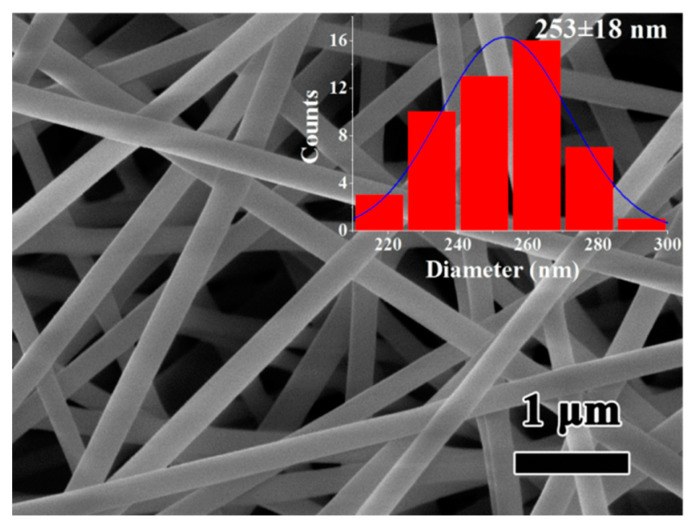
SEM image of polyvinyl alcohol (PVA) nanofibers (solution concentration 8%, applied electric field 17 kV, feed rate 0.010 mL/min, tip-to-collector distance 9 cm, needle type 20G), the inset is the histogram of diameter distribution.

Figure 2 shows SEM image of neat PVA NFs, which are prepared at an appropriate electrospinning condition [19] of solution concentration 8%, applied electric field 17 kV, feed rate of 0.010 mL/min, tip-to-collector distance of 9 cm, and needle type 20G; the inset is the histogram of diameter distribution. It can be seen from Figure 2 that the surfaces of PVA NFs are smooth without any bead, droplet, or adhesion, and the fibers are straight along axial direction without bending or breakage. It indicates that the electrospinning parameters applied herein are appropriate for the preparation of high qualified PVA NFs. It also can be seen from Figure 2 that the average diameter of PVA NFs is 253 nm with an even distribution of ±18 nm.

**Figure 3:**
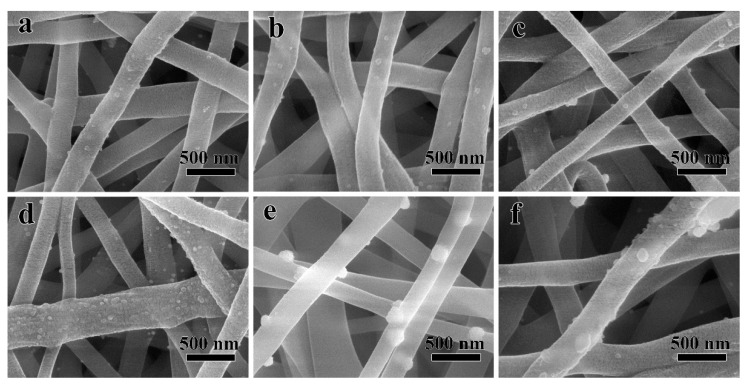
SEM images of Ag/PVA CNFs prepared with various concentrations of AgNO_3_, (**a**) 0.016, (**b**) 0.033, (**c**) 0.049, (**d**) 0.066, (**e**) 0.098 and (**f**) 0.132 mol/L.

After a rapid hydrothermal treatment process in EG, the Ag NPs could be decorated directly on the surface of PVA NFs, without the pre-grafting modification procedure. Figure 3 shows SEM images of Ag/PVA CNFs with various concentrations of AgNO_3_, (a) 0.016 (b) 0.033, (c) 0.049, (d) 0.066, (e) 0.098, and (f) 0.132 mol/L. Compared with Figure 2, it can be seen from Figure 3 that the morphology and average diameter of PVA NFs do not exhibit obvious changes. However, the surface of PVA NFs is not smooth anymore, and particles with brighter contrast can be observed after the solvothermal reaction, which is certainly due to the Ag NPs growth on the PVA NFs surface. It is worth noting that from Figure 3, it can be deduced that Ag NPs are adhered on the surface of PVA NFs, but not implanted inside the fibers. Since SEM images were taken at a relatively low acceleration voltage (i.e., 5 kV), the perpetration depth of electron beams is too limited to acquire Ag NPs images if they are embedded into the fibers [19]. Furthermore, the different brightness contrast of Ag NPs and PVA NFs in Figure 3 are attributed to difference between the electron transmittance of metal and polymer materials, the yield of secondary electron is enriched at the positions where Ag NPs emerged [30]. It also can be seen from Figure 3 that when the concentration of AgNO_3_ is 0.016 mol/L, the loading amount of Ag NPs is relatively low, and it does not increase remarkably until the concentration reaches 0.066 mol/L. As shown in Figure 3d, when the AgNO_3_ concentration is 0.066 mol/L, Ag NPs exhibit not only the highest loading densities, but also a narrow size distribution without aggregation. However, with the further increase of the AgNO_3_ concentration, the loading density does not improve accordingly, and the particles size become larger due to aggregation. This is because that when AgNO_3_ concentration is very high, the nucleation sites on PVA NFs surface are all occupied, the growth rate exceeds the nucleation rate. Numerous reports [31] have demonstrated that the antibacterial property improves with the decrease of nanoparticles size, so this indicates that 0.066 mol/L is the optimal concentration for preparation of Ag/PVA CNFs. Thus, the sample prepared at this condition was selected for further analysis of structure and antimicrobial activity. From the above-mentioned SEM analysis, it also can be found that the size and loading density of Ag NPs on PVA NFs can be facilely controlled through adjusting the concentration of AgNO_3_ in the solvothermal process, which provides an effective strategy to adjust the structure and properties of the as-fabricated Ag/PVA CNFs. Numerous reports have demonstrated that the particles size and morphologies of NPs are difficult to control if they are directly implanted into the NFs during the electrospun process [19,30], so the procedure proposed in this paper has the advantage of gaining a tunable structure and controllable property for composite electrospun NFs.

**Figure 4:**
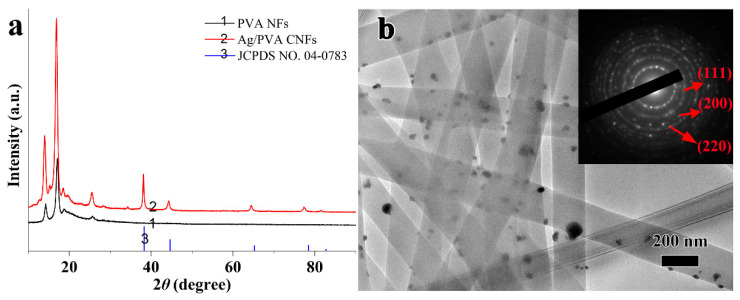
(**a**) XRD patterns of PVA NFs and Ag/PVA CNFs, (**b**) TEM image of Ag/PVA CNFs prepared with AgNO_3_ concentration at 0.066 mol/L, the inset is the SAED patterns of Ag NPs.

To further confirm the phase structure of the as-fabricated Ag/PVA CNFs, XRD characterization was conducted in this study. Figure 4a shows XRD patterns of PVA NFs and Ag/PVA CNFs. It can be seen from XRD pattern of PVA NFs that the diffraction peaks around 14.2° and 17.1° can be assigned to the diffraction from (100) and (001) lattice planes, respectively [32]. Furthermore, the weak band at 2θ of 18.8° can be assigned to a mixture diffraction from planes (101), (100), and (001). It also can be seen from Figure 4a that the diffraction peak at 2θ = 17.1° is enhanced, due to the rotation of PVA polymer molecular chains during the electrospinning, which has been explained in detail in our previous work [30]. Compared with XRD patterns of Ag/PVA CNFs and the neat PVA NFs, it is found that there are new diffraction peaks occurring at 2θ of 38.1°, 44.3°, 64.4°, 77.5°, and 81.5°, which are arising from the (111), (200), (220), (311), and (222) lattice planes diffraction of face-centered cubic (fcc) structured Ag (Joint Committee on Powder Diffraction Standards (JCPDS) No. 04-0783), respectively [33,34]. There are no diffraction peaks of Ag_2_O or other impurities occurring, indicating that Ag NPs are formed during the polyol solvothermal process and loaded on PVA NFs surfaces in situ. Meanwhile, the as-fabricated Ag/PVA CNFs also exhibit the diffraction peaks of PVA, which confirms the formation of composite nanofibers.

To gain a clearer morphology and composition information of Ag/PVA CNFs, TEM and SAED characterization were also employed. Figure 4b shows TEM image of Ag/PVA CNFs prepared at AgNO_3_ concentration of 0.066 mol/L, the inset is the SAED patterns of Ag NPs. It can be seen that the diameter distribution of PVA NFs is fairly uniform, with no obvious beads, droplets, or adhesions between NFs observed. PVA NFs are overlaid with random orientations to form a three-dimensional porous network structure. Furthermore, compared with Figure 2, the average diameter after solvothermal treatment does not show an obvious change, which indicates that the Ag NPs deposition process has no significant impact on the morphologies and diameter of matrix PVA NFs. However, a large number of quasi-spherical particles with a mean size of about 30 nm can be clearly identified on the surface of the PVA NFs. Obviously, this is attributed to the formation Ag NPs through the reduction of EG, since polymer NFs of PVA and metal NPs of Ag exhibit various contrast for the electron beam. Furthermore, the inset in Figure 4b of SAED reveals a ring-like diffraction pattern, and the rings could be indexed to the (111), (200) and (220) planes of the fcc crystalline lattice of Ag, which confirms the formation of Ag nanocrystals on the PVA NFs.

### 3.2. UV-vis and FTIR Spectra of Ag/PVA CNFs

**Figure 5:**
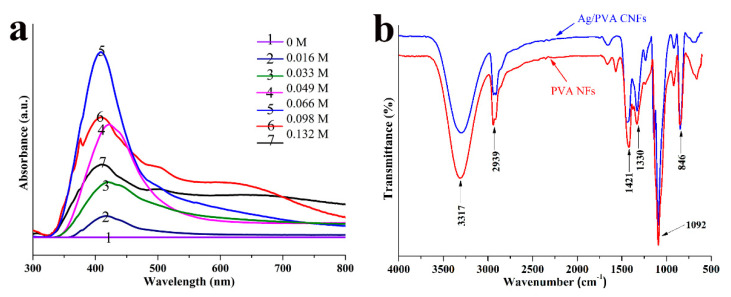
(**a**) UV-vis spectra of Ag/PVA CNFs prepared with different AgNO_3_ concentrations, (**b**) FTIR spectra of PVA NFs and Ag/PVA CNFs prepared with AgNO_3_ concentration of 0.066 mol/L.

It is well known that UV–vis spectroscopy is a powerful technique for the characterization of Ag NPs optical response, due to its characteristic surface plasmon resonance (SPR) bands, which are associated with particle peculiarity in terms of morphology, size, and distribution. Figure 5a shows UV-vis spectra of Ag/PVA CNFs prepared with different AgNO_3_ concentrations from 0.016 to 0.132 mol/L, and spectrum of 0 mol/L corresponding to the sample of neat PVA NFs. It is evident from Figure 5a that the neat PVA NFs do not exhibit any absorption bands, however, all the Ag/PVA CNFs prepared with different AgNO_3_ concentrations exhibit an obvious absorption band at 410–420 nm, which are attributed to the excitation of characteristic SPR for Ag NPs [35,36]. Moreover, the intensity, full width at half maximum (FWHM), and position of the absorption vary with the change of the AgNO_3_ concentration. As shown in Figure 5a, when the AgNO_3_ concentration is 0.066 mol/L, the sample exhibits a significant absorption band (#5 spectrum) with the maximum intensity and narrowest FWHM, compared with other UV-vis spectra. This implies that the Ag NPs loading density is the highest and the particles size distribution is the most even for this sample. Moreover, the band positions of other spectra are redshift, which indicates that the average particles size is the smallest for this sample. This result is exactly consistent with SEM observation. Thus, it is reasonable to conclude that 0.066 mol/L is the optimal AgNO_3_ concentration for the decoration of Ag NPs on PVA NFs surfaces, since the sample prepared in this condition has the narrowest particles size distribution and the highest loading density.

To gain a deeper insight into the interaction of Ag NPs with PVA NFs surface, FTIR spectroscopy is employed to investigate the chemical bonding states of samples. Figure 5b shows the FTIR spectra of PVA NFs and Ag/PVA CNFs prepared with AgNO_3_ concentration of 0.066 mol/L. It can be seen from the FTIR spectrum of PVA NFs that several absorption bands are present, which could be assigned to the following vibrational modes. The broad and intensive band centered at 3317 cm^−1^ is attributed to the stretching vibrations stretching of –OH, which are abundant in the segments of PVA polymers. Also, the sharp band at 1092 cm^−1^ and the shoulder band around 1330 cm^−1^ are arising from vibrations of –OH, in the models of in-plane bending and its coupling, respectively [30]. Meanwhile, the bands at 2939 cm^−1^ and 846 cm^−1^ can be assigned to the symmetric stretching and out-of-plane twisting vibration modes of –CH_2_– in polymer chains, respectively [37]. The band at 1421 cm^−1^ is attributed to the wagging vibrations of C–H [38]. Compared with the FTIR spectrum of neat PVA NFs, no obvious changes occur in the peak position for Ag/PVA CNFs. However, it should be pointed out that a change of ratio between the intensities of band at 1330 cm^−1^ and 1420 cm^−1^ occurs, which suggests that the interaction between Ag NPs and the matrix takes place over the O-H groups [39]. Thus, it is inferred that Ag NPs are formed by the reduction reaction of EG and loaded on PVA NFs through a chemical interaction between the reactive surface Ag atoms in NPs and oxygen atoms in hydroxyl groups.

### 3.3. Antibacterial Properties of Ag/PVA CNFs

The antibacterial activities of Ag/PVA CNFs were evaluated by the methods of absorption, turbidity, and growth curve using *E. coli* and *S. aureus* as target species, respectively. As partial results of absorption method, Figures S1 and S2 show photographs of survival colonies on the agar Petri dish treated by Ag/PVA CNFs prepared with different AgNO_3_ concentration of (a) neat PVA, (b) 0.016, (c) 0.033, (d) 0.049, (e) 0.066, (f) 0.098, and (g) 0.132 mol/L against *E. coli* and *S. aureus*, respectively. It can be seen from Figure S1 that there are a small number of colonies occurring on each agar Petri dish. The number of survival colonies decreased gradually, and then increased slightly with the increase of the AgNO_3_ concentration, indicating that the best antimicrobial activates of Ag/PVA CNFs are obtained when the concentration of AgNO_3_ is 0.066 mol/L. This sample possesses the narrowest Ag particles size distribution and the highest loading density, as reveled as the above-mentioned structural characterization. Comparing Figure S2 with Figure S1, the change tendency of survival colonies with AgNO_3_ concentration is as the same. However, there are no colonies that can be seen for the sample of Ag/PVA CNFs prepared with the AgNO_3_ concentration at 0.066 mol/L, as shown in Figure S2. Furthermore, all samples exhibit less discernible colonies against *S. aureus* than that of *E. coli*. It implies that the as-fabricated Ag/PVA CNFs exhibit a more significant inhibition effect to Gram-positive bacteria of *S. aureus* than Gram-negative bacteria of *E. coli*. This result is similar to that of our previous work [19].

Besides Figures S1 and S2, the results of adsorption test were also shown in the form of the antibacterial rate, which is calculated by specific equations described in our previous work [30], based on the numbers of bacteria which were measured by a colony counter (YLN-50A, Yalien Scientific Equipment Company, Beijing, China). Table 1 and Table 2 show the antibacterial test results by the adsorption method for Ag/PVA CNFs prepared with different concentrations of AgNO_3_ against *E. coli* and *S. aureus*, respectively. In these tables, the number of colonies was counted by using the colony counter, and the total numbers of the bacteria are products caudated by multiplying the colony on the dish, diluent ratio, the volume of eluent and coefficient. The detail calculation method can be found in the reference [30]. As for the antibacterial rate, it is calculated according to the following equation:

(1) $$\;X_{s}=\frac{M_{o}-M_{t}}{M_{o}}$$

where *X_s_* is antibacterial rate (%); *M_t_* is the total number of the bacteria of the testing sample; and *M_0_* is the total number of the bacteria of the control sample (cfu/mL). In this study, the control samples are the neat PVA NFs without Ag NPs loading. It can be seen from Table 1 and Table 2 that the total numbers of the bacteria treated with Ag/PVA CNFs are far smaller than that of neat PVA, which indicates that the loading of Ag NPs endows excellent bacterial inhibition to PVA NFs. For both the strains of *E. coli* and *S. aureus*, antibacterial rates increased gradually and decrease with the increasing of AgNO_3_ and reach the highest values of 98% and >99%, respectively. This confirms the appropriate AgNO_3_ concentration of 0.066 mol/L once again. Moreover, compared with the data shown in Table 1 and Table 2, it is evident that the as-fabricated Ag/PVA CNFs exhibit superior antibacterial activities against *S. aureus*. The reasons might be attributed to the PVA NFs itself possess bacteriostatic ability to some extent, and this effect is more pronounced for *S. aureus* than that of *E. coli.* From Table 1 and Table 2, it also can be concluded that the antibacterial activates of Ag/PVA CNFs can be easily adjusted by controlling the AgNO_3_ concentration.

**Figure 6:**
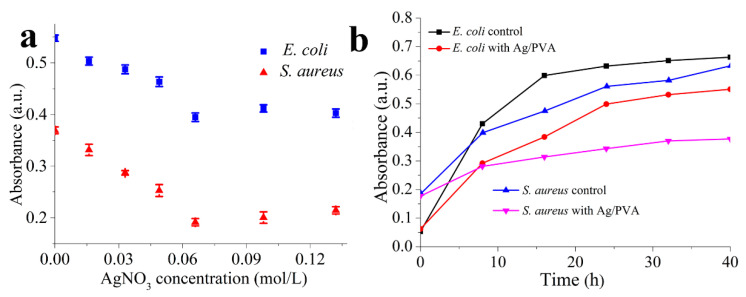
(**a**) Variation relationship between UV-vis absorbance and different concentrations of AgNO_3_ for Ag/PVA CNFs, (**b**) bacteria-growth curves of *E. coli* and *S. aureus* with and without Ag/PVA CNFs treatment.

The turbidity method is usually used for the evaluation of antibacterial activities; absorbance is positively related with the concentration of survival colonies in solution. Figure 6a shows the variation relationship between UV-vis absorbance and different concentrations of AgNO_3_ for Ag/PVA CNFs against *E. coli* and *S. aureus*, respectively. Regarding both *E. coli* and *S. aureus*, it is obvious that a minimum absorbance is present, indicating the most excellent antimicrobial activity, at the AgNO_3_ concentration of 0.066 mol/L. However, the absorbance should increase with the increase or decrease of the concentration. From the above-mentioned morphology and structure analysis, it is known that at this AgNO_3_ concentration, Ag NPs possess the smallest average particles size and distribution, accompanied by the highest loading density, which is responsible for the superior antimicrobial activity. Moreover, the absorbance of *S. aureus* is less than that of *E. coli* at the same concentration of AgNO_3_, which indicates once more the antibacterial ability of Ag/PVA CNFs is more significant against *S. aureus* than that of *E. coli*.

Drawing the bacterial growth curves presents a visual method to reflect the relationship of antibacterial effect with incubation time. Thus, this method is also employed in this study to reveal in depth the antibacterial properties of Ag/PVA CNFs. Figure 6b shows bacteria-growth curves of *E. coli* and *S. aureus* treated with Ag/PVA CNFs prepared with AgNO_3_ concentration of 0.066 mol/L, and the growth curves of strains without treatment of Ag/PVA CNFs as the control tests. As shown in Figure 6b, for the strains of *S. aureus*, bacteria without Ag/PVA CNFs treatment can be rapidly proliferated. However, there is a prominent inhibitory effect when Ag/PVA CNFs is added in the bacterial suspension. The absorbance of bacterial suspension treated with Ag/PVA CNFs is significantly lower than that of the control group, after cultivation in constant temperature and humidity condition for 8 h, indicating that Ag/PVA CNFs shows an obvious bacteriostatic effect on *S. aureus*. With the increasing of incubation time, the absorbance value of bacteria suspension containing Ag/PVA CNFs increases very slightly, suggesting that the antibacterial activities of Ag/PVA CNFs are maintained without significant loss throughout the measured time range (40 h in this case). A similar phenomenon is also observed for *E. coli* shown in Figure 6b. From the results of the growth curves test, it is suggested that the as-fabricated Ag/PVA CNFs exhibit not only excellent antibacterial activities, but also a certain antibacterial persistence.

The excellent antimicrobial activity is mainly attributed to the Ag NPs loading on the surface of PVA NFs. Although the precise action mechanism of Ag NPs against bacteria has not yet been accurately determined, the possible antibacterial action models can be proposed based on the antibacterial test results. Bacteria are inclined to be attached on the Ag/PVA CNFs, due to the positive charge of Ag NPs and the rugged surface of the fibers. Then, detached Ag NPs or released Ag+ penetrate the cell of bacteria to cause further damage [40,41,42]. In addition, the possible antibacterial mechanism of Ag/PVA CNFs also involves superabundant reactive oxygen species (ROS) formation in bacterial cells [43,44,45]. Thus, the regulation of Ag NPs size, distribution and loading density is of great importance to the antibacterial properties of the as-fabricated Ag/PVA CNFs.

## 4. Conclusions

To summarize, we have successfully fabricated Ag NPs surface decorated PVA composite nanofibers through a convenient, cost-effective, and polymer grafting free strategy by combining electrospinning and solvothermal techniques. The as-fabricated Ag/PVA CNFs were well characterized by techniques of SEM, TEM, SAED, XRD, UV-vis, and FTIR. The particles size, distribution, and loading density were easily tailored by adjusting the AgNO_3_ concentration, and 0.066 mol/L was found to be an optimal condition for the morphological and structural adjustment. In addition, the possible growth process and interaction of Ag NPs with PVA NFs are presented. It was inferred that the combination of Ag NPs and PVA matrix is through a chemical interaction between the reactive surface Ag atoms in NPs and oxygen atoms in hydroxyl groups. The antimicrobial properties were investigated by the methods of absorption, turbidity, and growth curves against the bacteria of *E. coli* and *S. aureus*. The results show that the as-fabricated Ag/PVA CNFs exhibit excellent antimicrobial activities, especially for the sample prepared with an AgNO_3_ concentration of 0.066 mol/L. Its antibacterial rate reaches 98% and 99% for *E. coli* and *S. aureus*, respectively. Furthermore, the antibacterial ability of Ag/PVA CNFs is more significant against *S. aureus* than that of *E. coli*. Meanwhile, it also exhibits antibacterial persistence to a certain extent. This result indicates that the antibacterial activities also can be controlled easily by adjusting Ag NPs size and loading density on the PVA NFs. Combined with the advantages of convenient preparation, controllable structure, and excellent antibacterial properties, the as-fabricated Ag/PVA CNFs have potential applications in many fields such as nonwoven fabrics, surgical dressing, and packaging films.

## Figures and Tables

**Table 1:** Antibacterial test results by adsorption method for Ag/PVA CNFs prepared with different concentrations of AgNO_3_ against *E. coli*.

AgNO_3_ Concentration (mol/L)	Diluent Ratio and Colony Count			Total Numbers of the Bacteria (CFU/mL)	Antibacterial Rate (%)
	10	10^2^	10^3^		
Neat PVA	Innumerable	59	7	1,180,000	——
0.016	Innumerable	32	3	640,000	46
0.033	Innumerable	18	1	360,000	70
0.049	32	4	0	64,000	95
0.066	9	2	0	20,000	98
0.098	Innumerable	12	0	24,000	98
0.132	Innumerable	15	0	30,000	97

**Table 2:** Antibacterial test results by adsorption method for Ag/PVA CNFs prepared with different concentrations of AgNO_3_ against *S. aureus*.

AgNO_3_ Concentration (mol/L)	Diluent Ratio and Colony Count			Total Numbers of the Bacteria (CFU/mL)	Antibacterial Rate (%)
	10	10^2^	10^3^		
Neat PVA	Innumerable	14	2	280,000	——
0.016	Innumerable	8	0	160,000	43
0.033	21	1	0	42,000	85
0.049	8	0	0	16,000	94
0.066	2	0	0	0	>99
0.098	15	1	0	20,000	93
0.132	18	2	0	40,000	85

## Supplementary Materials

The following are available online at https://www.mdpi.com/2073-4360/12/11/2486/s1, Figure S1: Photographs of survival colonies for Ag/PVA CNFs against *E. coli*, Figure S2: Photographs of survival colonies for Ag/PVA CNFs against *S. aureus*.
