# Supplementary material for: A Facile Method for the Fabrication of Silver Nanoparticles Surface Decorated Polyvinyl Alcohol Electrospun Nanofibers and Controllable Antibacterial Activities
Source: Polymers (Basel). 2020 Oct 26;12(11):2486. doi: 10.3390/polym12112486 (PMC7693976; doi:10.3390/polym12112486)
Supplement: Supplementary file 1 [file polymers-12-02486-s001.pdf]

polymers

Supplementary Material of

A Facile Method for the Fabrication of Silver Nanoparticles Surface Decorated Polyvinyl Alcohol Electrospun Nanofibers and Controllable Antibacterial Activities

Yan Yang [1], Zhijie Zhang [2], Menghui Wan [1], Zhihua Wang [3,*], Xueyan Zou [1], Yanbao Zhao [1] and Lei Sun [1,*]

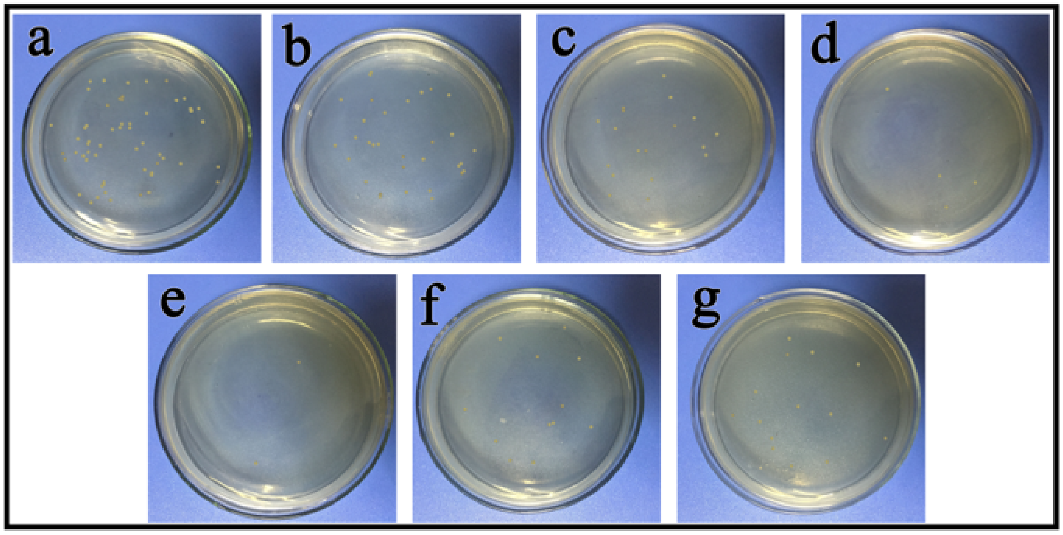

Figure 1. Photographs of survival colonies on the agar Petri dish evaluated by the absorption method for Ag/PVA CNFs prepared with different $AgNO_3$ concentration of (**a**) neat PVA, (**b**) 0.016, (**c**) 0.033, (**d**) 0.049, (**e**) 0.066, (**f**) 0.098, and (**g**) 0.132 mol/L against *E. coli.*

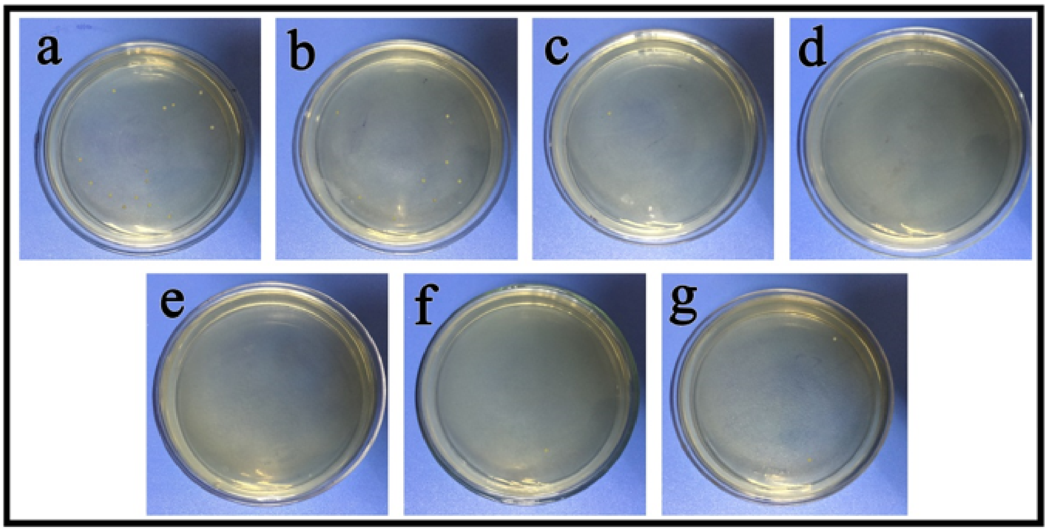

Figure 2. Photographs of survival colonies on the agar Petri dish evaluated by the absorption method for Ag/PVA CNFs prepared with different $AgNO_3$ concentration of (**a**) neat PVA, (**b**) 0.016, (**c**) 0.033, (**d**) 0.049, (**e**) 0.066, (**f**) 0.098, and (**g**) 0.132 mol/L against *S. aureus*.

Publisher's Note: MDPI stays neutral with regard to jurisdictional claims in published maps and institutional affiliations.

© 2020 by the authors. Submitted for possible open access publication under the terms and conditions of the Creative Commons Attribution (CC BY) license (http://creativecommons.org/licenses/by/4.0/).
